# Sulindac as a precision microRNA modulator in early-stage K-Ras-driven oncogenesis

**DOI:** 10.1038/s41420-025-02870-6

**Published:** 2025-11-26

**Authors:** Christos Adamopoulos, Kostas A. Papavassiliou, Athanasios G. Papavassiliou

**Affiliations:** 1 Department of Biological Chemistry, Medical School, National and Kapodistrian University of Athens, Athens, Greece; 2 Department of Oncological Sciences, Icahn School of Medicine at Mount Sinai, New York, NY USA; 3 First University Department of Respiratory Medicine, ‘Sotiria’ Chest Hospital, Medical School, National and Kapodistrian University of Athens, Athens, Greece

**Keywords:** Tumour biomarkers, Cancer therapy

The prevention of cancer through pharmacological intervention has long been a tantalizing yet challenging objective. Non-steroidal anti-inflammatory drugs (NSAIDs) have consistently emerged as promising candidates, supported by epidemiological and clinical studies linking their use with reduced incidence of colorectal and other cancers [1]. Their effects extend beyond cyclo-oxygenase (COX) blockade, implicating COX-independent mechanisms such as modulation of apoptosis, signal transduction, immune responses, and even microRNA (miRNA) regulation [2, 3]. This expanded perspective has revived attention to NSAIDs as versatile agents in cancer chemoprevention, especially as resistance and heterogeneity limit targeted regimens. To this end, sulindac, a prototypical NSAID, has demonstrated preventive and therapeutic activity in breast, pancreatic, uterine, and colorectal cancers [4–7]. The recent study by Liang et al. [8] provides crucial mechanistic insight, revealing that sulindac sulfide (SS), the active metabolite of sulindac, restores tumor-suppressive miRNA lethal-7b (Let-7b) to disrupt the Kirsten rat sarcoma viral oncogene homolog (K-Ras)/extracellular signal-regulated kinase (ERK)/lin-28 RNA-binding post-transcriptional regulator B (LIN28B) feedback loop, reframing sulindac as a precision modulator of oncogenesis.

**Fig. 1:**
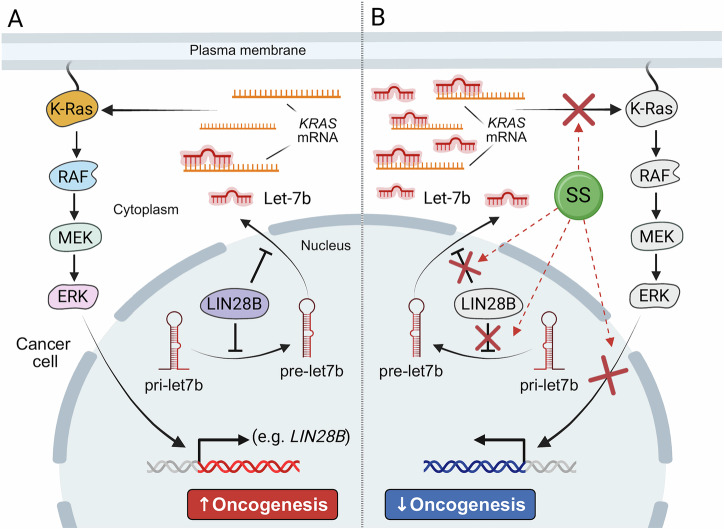
Restoration of Let-7b by sulindac sulfide (SS) attenuates K-Ras-driven tumorigenesis. **A** In the absence of SS, mutant-K-Ras activates the RAF−MEK−ERK signaling pathway, leading to phosphorylation and activation of ERK. Activated ERK induces the expression of genes that promote oncogenic transformation. Consequently, the expression of the RNA-binding protein LIN28B is induced, which inhibits the maturation of pri-let-7b into pre-let-7b in the nucleus and into mature microRNA Let-7b in the cytoplasm. Reduced Let-7b levels relieve repression of K-Ras mRNA, reinforcing the oncogenic loop and promoting tumorigenesis. **B** In the presence of SS, ERK phosphorylation and LIN28B expression are inhibited, thereby restoring Let-7b biogenesis. Elevated levels of Let-7b bind K-Ras mRNA and suppress its expression, breaking the oncogenic loop and reducing transformation. ERK extracellular signal-regulated kinase, K-Ras Kirsten rat sarcoma viral oncogene homolog, Let-7b lethal-7b, LIN28B lin-28 RNA-binding post-transcriptional regulator B, MEK mitogen-activated protein kinase kinase, pre-let-7b precursor let-7b, pri-let-7b primary let-7b transcript, RAF rapidly accelerated fibrosarcoma, SS sulindac sulfide. Created in BioRender (https://BioRender.com/ks3yqge).

NSAIDs have long been recognized for their chemopreventive effects in colorectal cancer (CRC), largely attributed to COX inhibition [1]. Nevertheless, evidence highlights COX-independent pathways influencing apoptosis and tumorigenesis [2]. Meta-analyses reinforce these notions, associating NSAIDs use with reduced metastasis and improved survival [3]. Liang et al. [8] widen that by showing that SS enhances Let-7b expression, a miRNA that directly suppresses K-Ras [9] (Fig. 1B). Their discovery is of vital importance as K-Ras remains one of the most frequently mutated oncogenes across cancers. These findings align with sulindac’s immune-modulatory effects in breast cancer [4] and its chemopreventive efficacy in murine models [5]. Together, they redefine sulindac as a multi-target agent acting at both inflammatory and post-transcriptional levels.

Liang et al. [8] describe a feedback circuit whereby K-Ras activation facilitates ERK phosphorylation, inducing the expression of LIN28B, which suppresses Let-7b biogenesis (Fig. 1A). This molecular circuitry amplifies oncogenic signaling. By reducing phosphorylated ERK (p-ERK) and LIN28B, SS breaks this cycle, reinstating Let-7b and attenuating transformation (Fig. 1B). Knockdown experiments confirmed specificity: loss of Let-7b abolished SS’s effect, while Let-7g (another member of the Let-7 family) deprivation had no impact. Such precision suggests potential biomarker applications, where Let-7b could predict sulindac responsiveness. Notably, restoring tumor-suppressive miRNAs may be as valuable as targeting kinases, offering an indirect but powerful way to control K-Ras activity.

Sulindac has been central in chemoprevention, particularly in familial adenomatous polyposis (FAP), where it reduces adenoma burden [10]. The regulatory role of Let-7b axis adds mechanistic depth to these observations and is in accordance with evidence of sulindac suppressing adenoma formation in murine models [5, 11]. Furthermore, sulindac was able to reverse resistance, which was mediated by the 15-hydroxyprostaglandin dehydrogenase (15-PGDH; a key enzyme that catalyzes the conversion of PGE2 to biologically inert compounds) pathways [12]. These insights emphasize the importance of characterizing patients for both K-Ras mutational status and Let-7b expression, integrating NSAIDs with other targeted treatments in patients with inherited risk [10]. In this vein, sulindac could serve as a biomarker-driven intervention tailored to the specific molecular context.

Targeted therapies focusing solely on K-Ras inhibition are unlikely to achieve durable responses without combinatorial strategies. Preclinical models imply that combining sulindac with Wingless/Integrated (Wnt) signaling pathway inhibitors significantly decreases adenoma formation in mice [13], whereas sulindac derivatives have displayed synergistic effects with chemotherapy in pancreatic cancer, overcoming resistance to gemcitabine and nanoparticle albumin-bound paclitaxel (nab-paclitaxel) [6]. These findings suggest a dual role for sulindac in both chemoprevention and treatment, rendering it relevant across the cancer continuum. Beyond COX inhibition, novel sulindac analogs such as sulindac sulfide amide (SSA; a N,N-dimethylethyl amine derivative of SS) exhibit anti-tumor activity through inhibition of the Akt (PKB)/mechanistic target of rapamycin (mTOR) pathway [14]. These combination regimens can provide opportunities to optimize Let-7b regulation and, at the same time, minimize toxicity. Remarkably, given sulindac’s demonstrated immune-modulatory properties [4], another promising avenue is to explore co-targeting strategies with immunotherapy, where sulindac may boost immune surveillance and responsiveness to checkpoint blockade. Therefore, a favorable strategy would be to evaluate sulindac in conjunction with the newly developed direct K-Ras inhibitors, such as the allele-specific K-Ras^G12C^ inhibitors, or other mitogen-activated protein kinase (MAPK) pathway inhibitors, to foster efficacy and overcome resistance mechanisms. These multifaceted strategies coordinate with broader perspectives on chemoprevention and collectively position sulindac as a “tool” for rational repurposing across the tumor spectrum [15].

K-Ras mutational heterogeneity presents a significant challenge for sulindac-based chemoprevention. Rice et al. [11] demonstrated in colon cancer murine models that specific K-Ras mutations, including the prevalent K-Ras^G12D^, reduce sulindac effectiveness, indicating that mutational context is a critical determinant of therapeutic response and underscores that variability of NSAIDs responsiveness is evident in preclinical settings. Although NSAIDs exhibit protective effects against metastasis [3], the underlying molecular mechanisms require further investigation. Incorporating mutational profiling, miRNA signatures, and pharmacodynamic biomarkers will be necessary to advance the clinical application of sulindac.

Sulindac and related NSAIDs display anti-tumor therapeutic potential beyond CRC. Novel non-COX inhibitory derivatives of sulindac hamper cell proliferation and invasion in uterine carcinoma and reduce mammary tumorigenesis in vivo [7]. Additionally, sulindac itself has been shown to augment anti-tumor immunity in breast cancer [4]. Evidently, NSAIDs can control inflammation beyond COX, affecting redox and resolution phase pathways [2]. The above observations are in agreement with those of Liang et al., which show that SS restores the regulatory balance in K-Ras feedback circuits by modulating the Let-7b miRNA [8]. Collectively, sulindac emerges as a wide-range modulator of tumor progression.

Liang et al. reinvent sulindac as a miRNA-modulating chemopreventive agent, which can impede K-Ras-mediated signaling via Let-7b restoration [8]. Their findings re-evaluate NSAIDs’ role from broad chemopreventive tools to precision modulators of oncogenic signaling networks. Future work should assess K-Ras mutation-specific efficacy and in vivo potency and appraise combination strategies and clinical biomarkers such as Let-7b. Towards this direction, optimizing sulindac derivatives could strengthen effectiveness, specificity, and safety. Ultimately, sulindac’s repurpose within precision oncology offers a chance to intercept tumorigenesis at its earliest, most reversible stages.
